# Effect of Osmotic Pressure on the Stability of Whole Inactivated Influenza Vaccine for Coating on Microneedles

**DOI:** 10.1371/journal.pone.0134431

**Published:** 2015-07-31

**Authors:** Hyo-Jick Choi, Jae-Min Song, Brian J. Bondy, Richard W. Compans, Sang-Moo Kang, Mark R. Prausnitz

**Affiliations:** 1 School of Chemical and Biomolecular Engineering, Georgia Institute of Technology, Atlanta, Georgia, United States of America; 2 Department of Chemical and Materials Engineering, University of Alberta, Edmonton, Alberta, Canada; 3 Department of Global Medical Science, Sungshin Women's University, Seoul, Korea; 4 Department of Microbiology and Immunology, Emory University School of Medicine, Atlanta, Georgia, United States of America; 5 Center for Inflammation, Immunity, & Infection and Department of Biology, Georgia State University, Atlanta, Georgia, United States of America; Icahn School of Medicine at Mount Sinai, UNITED STATES

## Abstract

Enveloped virus vaccines can be damaged by high osmotic strength solutions, such as those used to protect the vaccine antigen during drying, which contain high concentrations of sugars. We therefore studied shrinkage and activity loss of whole inactivated influenza virus in hyperosmotic solutions and used those findings to improve vaccine coating of microneedle patches for influenza vaccination. Using stopped-flow light scattering analysis, we found that the virus underwent an initial shrinkage on the order of 10% by volume within 5 s upon exposure to a hyperosmotic stress difference of 217 milliosmolarity. During this shrinkage, the virus envelope had very low osmotic water permeability (1 – 6×10^−4^ cm s^–1^) and high Arrhenius activation energy (*E*_a_ = 15.0 kcal mol^–1^), indicating that the water molecules diffused through the viral lipid membranes. After a quasi-stable state of approximately 20 s to 2 min, depending on the species and hypertonic osmotic strength difference of disaccharides, there was a second phase of viral shrinkage. At the highest osmotic strengths, this led to an undulating light scattering profile that appeared to be related to perturbation of the viral envelope resulting in loss of virus activity, as determined by *in vitro* hemagglutination measurements and *in vivo* immunogenicity studies in mice. Addition of carboxymethyl cellulose effectively prevented vaccine activity loss *in vitro* and *in vivo*, believed to be due to increasing the viscosity of concentrated sugar solution and thereby reducing osmotic stress during coating of microneedles. These results suggest that hyperosmotic solutions can cause biphasic shrinkage of whole inactivated influenza virus which can damage vaccine activity at high osmotic strength and that addition of a viscosity enhancer to the vaccine coating solution can prevent osmotically driven damage and thereby enable preparation of stable microneedle coating formulations for vaccination.

## Introduction

This study focused on virus particle stability in hyperosmotic conditions is motivated by the need to stabilize vaccines during preparation of microneedle (MN) patches. Previous studies have shown that MN patches coated with influenza and other vaccines provide effective skin vaccination that generates protective immune responses that are at least as potent as conventional intramuscular or subcutaneous vaccination [1–3]. Advantages of MNs include the potential for vaccine dose-sparing and improved immunogenicity via alternative administration routes [4–7]. Because solid MN vaccines provide a promising platform for long-term stability and maintenance of protective immunogenic potency, considerable efforts have been devoted to the fabrication of various types of MNs and demonstrating their *in vivo* benefits. However, there has been little mechanistic study done on the initial activity loss of the vaccine during the MN preparation process [8]. Among many factors involved in this problem (e.g., phase transformation, dehydration effects, interaction between vaccine and substrate, osmotic stress, pH change, etc.) we hypothesize that osmotic stress is a significant underlying problem for MN coating with enveloped vaccines/viruses.

Enveloped biological systems are subjected to osmotic stress during drying processes and in high osmotic strength solutions. Osmotic pressure, arising from osmolarity differences across a semipermeable lipid membrane, induces swelling or shrinkage of biological systems as a result of water/osmolyte transportation [9]. The result of osmotic gradient-driven movement of water is morphological and these changes can influence the functional integrity and physiological processes of the organisms [10]. Most microorganisms, as well as human/animal/plant cells, maintain osmotic homeostasis through synthesis of osmoprotective molecules and/or osmo-sensory/regulatory membrane proteins [11,12]. However, the absence of osmoregulatory water channels such as aquaporins makes enveloped viruses more vulnerable to osmotic damage [13]. For example, Marek’s disease vaccine demonstrated a significantly lowered viability at an elevated osmolarity of 475 mOsm [14]. Therefore, the possible loss of functional activity associated with osmotic pressure is an issue that needs to be addressed when developing viral vaccine formulations.

Previous work has shown that spray-dried *Mycobacterium smegmatis*, Bacillus Calmette—Guérin (BCG), and influenza vaccines were not damaged during drying due to the absence of osmolytes (salts, cryoprotectant) in the process [15,16]. Unfortunately, a spray drying method is not compatible with typical MN fabrication processes. As another approach, previous studies have shown that cryoprotectants such as non-reducing disaccharides can prevent fusion of liposomes and leakage of entrapped materials from their cytoplasm, and preserve proteins in an active form during drying [17,18]. However, our previous work has shown that sugars can crystallize during long-term storage and thereby damage coated vaccine [19]. Despite stability issues during the initial drying and long-term storage, cryoprotectants have become a common component in preserving biomolecules during dehydration. As a result, non-reducing disaccharides, specifically trehalose, are a major component of many MN formulations [20–22]. However, such disaccharides, when present at such high concentrations also generate osmotic pressure on the vaccine during drying. Thus, the osmotic pressure increase caused by the addition of trehalose to the coating formulation could potentially offset any protective effects it may offer.

Studies on the morphological changes of vaccines in the presence of osmotic gradients are essential to understand the role of osmotic stress in vaccine stability. Osmotic pressure most likely induces morphological changes such as shrinkage, fission, swelling, and fusion of viral membranes, as seen in other enveloped organisms. For instance, events such as the formation of daughter cells in unilamellar liposomes [23] were also observed in biological systems such as *E*. *coli* when subjected to hypertonic osmotic conditions [24]. In the case of algae, higher plants, and Gram-negative bacteria, high osmotic pressure is needed to pull the cytoplasmic membrane away from their rigid cell walls and induce plasmolysis. On the other hand, in eukaryotic cells, plasmolysis occurs at relatively low hypertonic stress levels [25–27]. From these examples, it is evident that understanding a membrane’s structure is a critically important step in understanding how it will respond to osmotic stress.

Although there has been much research on the effects of osmotic pressure on the growth and viability of viruses (influenza virus [28], polio virus [29], Sindbis virus [30], and herpes virus [31]) and on the virus-cell fusion process [32–34], very little is known about osmotic effects on enveloped virus/vaccine itself [35]. A lack of general knowledge about the osmotic response of enveloped viruses is a major obstacle in predicting the stability of vaccine under various conditions. Understanding osmotic pressure-dependant stability of virus/vaccine coated on MNs is important to future clinical applications because it is directly related to the efficacy of vaccination. Therefore, one of our goals is to understand the fundamental osmotic behavior of the enveloped virus/vaccine.

In this work, osmotic shrinkage of live and inactivated H1N1 influenza A virus was observed using stopped-flow light scattering (SFLS) analysis at different osmotic gradients. Results were correlated with the infectivity and hemagglutination (HA) activity of the live and inactivated viruses, respectively. Based on the assumption that osmotic pressure-induced morphological change is a key factor affecting vaccine stability, vaccine-coating formulations were modified by increasing viscosity to delay the viral shrinkage rate and to minimize viral membrane perturbation. To validate this idea, the *in vitro* stability of virus/vaccine in coating formations with and without viscosity enhancer were examined in both liquid and dry states and their activity differences were compared. Furthermore, the effects of viscosity enhancers in the vaccine formulation were investigated *in vivo* using vaccine-coated MNs.

This research shows that enveloped influenza vaccine in hypertonic solutions experiences step-wise morphological changes: a rapid initial shrinkage together with membrane perturbations following a secondary shrinkage. This shrinkage was found to be related to vaccine activity loss. Our data suggest that osmotic pressure-induced vaccine destabilization in both liquid and dry states can be prevented by adding a viscosity enhancer to the vaccine formulation. This research is expected to contribute to improving our understanding of the behavior of enveloped viruses under osmotic stress as well as the development of immunogenic influenza vaccine-coated MNs.

## Materials and Methods

### Ethics Statement

All animal care and procedures presented in this work were approved by the Emory University Institutional Animal Care and Use Committee (IACUC) review board and conducted in accordance with the guidelines of the Emory University IACUC. Emory IACUC operates under the federal Animal Welfare Law (administered by the USDA) and regulations of the Department of Health and Human Services.

### Virus preparation

A/PR/8/1934 influenza virus was prepared using hen’s eggs and purified as described previously [36]. Inactivation with formalin was followed by plaque assays on Madin-Darby canine kidney (MDCK) cells to confirm virus inactivation. Both live and inactivated virus vaccines were used to investigate the effects of osmotic pressure on their stability.

### Osmotic shrinking kinetics of virus

Osmotic leakage properties of the inactivated influenza virus were analyzed by SFLS. The osmotic behavior of the virus was characterized by recording light scattering after a rapid mixing of inactivated influenza virus stock in DPBS with an equal volume of a separate solution of trehalose-dihydrate, sucrose, or NaCl (Sigma Aldrich, St. Louis, MO) in sterile Dulbecco’s phosphate-buffered saline (DPBS, Mediatech, Manassas, VA; pH adjusted to 7.5).

Osmolarities of all solutions were measured using a vapor pressure osmometer (Vapro 5520, Wescor Inc., Logan, UT) with reference to Wescor osmolarity standards. Several osmolarities were tested to compare the effect of osmotic gradient on water permeability of the virus and to monitor its shrinking behavior. This was performed with a stopped-flow spectrometer (MOS-200/M spectrometer, SFM-20; Bio-Logic USA, Knoxville, TN) at a flow rate of 7 mL s^–1^ (injection volume 66 μL) at 4°C. The excitation wavelength was set at 546 nm using a 150 watt Xe light source and monochromator (f/3.5 grating).

The osmotic water permeability coefficient of viruses (*P*_f_) was calculated using the equation,

$$P_{\text{f}}=k\cdot V_{\text{0}}/A\cdot V_{\text{w}}\cdot \Delta {\text{C}}_{\text{os}}$$

where *k* = rate constant, *V*_0_ = initial virus volume, *A* = initial virus surface area, *V*_w_ = molar volume of water, and ΔC_os_ = osmolarity difference across the viral envelope [37]. Initial virus size was measured by transmission electron microscopy (TEM, 100 kV) analysis after negative staining using phosphotungstic acid (pH = 7.0, Electron Microscopy Sciences, Hatfield, PA) [19]. A virus volume of 6.06 × 10^−16^ cm^3^ and a virus surface area of 3.46 × 10^−10^ cm^2^ in iso-osmotic condition were determined in this way and used for calculations. Values of *k* were obtained from the single exponential curve fitting of the light scattering spectra using Bio-Kine 32 V4.46 software (Bio-Logic USA).

The osmolarity of viral cytoplasm was determined to be C_os_ ~ 300 mOsm via measurements of its equilibrium osmolarity with outer medium to show iso-osmotic SFLS response, i.e. no light scattering intensity change. This allowed calculation of the osmotic gradient applied to the virus.

Temperature-dependent water permeability coefficient of the virus membrane was measured at a hyperosmotic difference of ΔC_os_ = 304 mOsm using trehalose (e.g., virus stock mixed with 600mM trehalose solution in DPBS). Measurements were made at six temperatures (4, 11, 18, 25, 32, and 40°C) maintained using a recirculating water bath.

Under the assumption that the relationship between SFLS light scattering and volume is the same for inactivated influenza virus and liposomes, the time-course of viral volume change was calculated following a method reported previously [38]. Briefly, the size change of liposomes (made of egg phosphatidylcholine, Avanti Polar Lipids, Alabaster, AL) was measured using dynamic light scattering (DLS), and SFLS was used at various osmotic gradients to relate the change in scattered light intensity to viral volumetric change.

### *In vitro* vaccine stability tests

To study the effects of osmotic pressure on virus activity, 2 μg (1 μg μL^–1^) of the live or inactivated influenza virus in DPBS was mixed with an equal volume of trehalose solution to apply a hyperosmotic difference of ΔC_os_ = 217, 420, 682, and 1351 mOsm in the same manner as for osmotic shrinkage experiments. That is, an osmotic strength difference of 217, 420, 682, or 1351 ΔmOsm was achieved by mixing virus stock with a 450, 800, 1200, and 2000 mM trehalose solution in DPBS, respectively. Virus-trehalose mixtures were incubated at 4°C in a sealed container to prevent drying-induced activity changes. They were subsequently diluted with DPBS after 10 s, 1 min, 5 min, 10 min, and 30 min to remove further application of the osmotic pressure. Over the course of incubation time, activity of the virus at each osmotic gradient was assessed by measuring HA activity. For HA titer measurements, a serial dilution of the sample was mixed with a 0.7% suspension of chicken red blood cells (Lampire Biological Laboratories, Pipersville, PA). HA activity of the samples at each condition was calculated relative to that of virus or inactivated virus in iso-osmotic condition (C_os_ = 300 mOsm, pH 7.4). All experiments were performed at 4°C to eliminate the temperature effect on the remaining HA activity.

To assess the effects of viscosity on the stability of the virus against osmotic stress, carboxymethyl cellulose sodium salt (Sigma Aldrich; abbreviated as CMC) was added to the trehalose solution as a viscosity enhancer. Over time, the HA activity change of a mixture composed of trehalose (ΔC_os_ = 682 mOsm), 0.5% w/v CMC, and inactivated influenza virus in DPBS was tested by measuring HA titer. HA titer measurements were performed on inactivated influenza virus (1 μg) in trehalose solution (ΔC_os_ = 217, 420, 682, and 1351 mOsm) with 0.5% w/v CMC after incubation for 30 min.

To investigate the effects of osmotic pressure on the stability of the live virus, viral infectivity was assayed by reading viral plaque forming units (PFU) from live influenza virus both with and without viscosity enhancer. Live influenza virus was mixed with trehalose only (ΔC_os_ = 682 mOsm) and trehalose (ΔC_os_ = 682 mOsm) plus 0.5% w/v CMC. Samples were incubated for 30 min at 4°C. Live influenza virus in iso-osmotic condition was used as a control. Viral titers were determined by counting the number of plaques formed on MDCK cells.

### Effect of viscosity on the stability of virus upon drying

To investigate the effects of viscosity on viral stability during drying, remaining HA activity of both live and inactivated viruses was tested using virus-embedded coatings on a titanium (Ti) plate, which is the same material and surface on which microneedle coatings are performed. Two different coating formulations were used for stability tests: trehalose (ΔC_os_ = 682 mOsm) solution; and trehalose (ΔC_os_ = 682 mOsm) plus 0.5% w/v CMC solution. It should be noted that the addition of CMC in the formulation had no detectible effect on the osmolarity of the solutions. One microgram of the live or inactivated influenza virus in these formulations was coated on a Ti plate at room temperature. The Ti plate was cleaned using acetone, methanol, and isopropanol (Sigma Aldrich), followed by plasma cleaning (PDC-32G, Harrick Plasma, Ithaca, NY) prior to use. The effects of viscosity enhancers on the stability of inactivated influenza virus were further tested using vaccine coating formulations composed of trehalose plus xanthan gum (Sigma Aldrich) or sodium alginate (Sigma Aldrich). For equal comparison, the concentrations of xanthan gum and sodium alginate were adjusted to have the same viscosity (i.e., 4.5 cP) as the trehalose plus 0.5% CMC formulation (0.075% for xanthan gum and 0.3% for sodium alginate). Coatings were air-dried at ambient conditions for 24 h and their remaining activity was assessed by HA titer measurement. HA titers of live and inactivated influenza virus in iso-osmotic condition at 4°C were used as controls of virus coatings. For HA titer measurement, dried coating samples were reconstituted in 200 μL of DPBS overnight at 4°C. Viscosity of the coating formulations was measured using glass capillary viscometers following the manufacturer’s protocol (Cannon Instrument, State College, PA). Viscosity was determined in triplicate.

### Fabrication of vaccine-coated MNs

To further evaluate the effects of viscosity on osmotic stress-induced viral instability, *in vivo* experiments were performed using two different kinds of inactivated influenza virus-coated MNs: virus coating with trehalose (ΔC_os_ = 682 mOsm) solution; and virus coating with trehalose (ΔC_os_ = 682 mOsm) plus 0.5% w/v CMC solution. MN arrays of five in-plane microneedles were fabricated by lithographic masking followed by wet etching of Ti sheets (needle dimension: 750 μm in length and 200 μm in width), as described previously [39]. MNs were cleaned following the same cleaning procedure as above for the Ti plates. MNs were coated using a dip-coating apparatus, as described elsewhere [39] and air-dried for 24 h at ambient conditions. Virus-coated MNs were imaged using optical microscopy (SZX12, Olympus America, Center Valley, PA) with a CCD camera (RT Slider, Diagnostic Instruments, Sterling Heights, MI).

### Immunization of mice

Female inbred BALB/c mice (Harlan Laboratories, Indianapolis, IN) 6–8 weeks of age were immunized once with inactivated influenza virus. Dried virus-coated MNs were reconstituted in DPBS for intramuscular immunization. Four groups of mice (6 mice per group) were immunized with 0.2 μg of the protein via intramuscular route: naïve (negative control), virus in iso-osmotic buffer solution at 4°C (positive control), virus in trehalose (ΔC_os_ = 682 mOsm) solution coated onto microneedles and then reconstituted, virus in trehalose (ΔC_os_ = 682 mOsm) solution containing 0.5% w/v CMC coated onto microneedles and then reconstituted. Protein concentrations were calculated using the bicinchoninic acid assay (BCA protein assay kit, Thermo Fischer scientific, Waltham, IL) with bovine serum albumin as a standard. To avoid interference from the coating materials, the concentration of coating excipients was maintained to be the same between the reconstituted virus solution from MNs and bovine serum albumin standard samples.

### Antibody responses

Serum samples were collected on the second week following immunization. Influenza virus-specific IgG antibody were measured using enzyme-linked immunosorbent assay (ELISA) plates coated with inactivated influenza virus and by goat anti-mouse IgG-specific secondary antibodies (Horseradish Peroxidase Conjugate, SouthernBiotech, Birmingham, AL) [40]. Optical densities were measured at 450 nm from the above-mentioned four groups.

### Statistical analysis

All parameters were recorded for individuals within all groups. Multiple conditions were compared using Student's *t*-test and general linear model (ANOVA), and *P* value of less than 0.05 was considered to be significant. In some cases, mean values were compared to validate the results.

## Results

### Permeability of inactivated influenza virus under osmotic stress

To understand the effects of osmotic stress on influenza virus stability, we first determined viral membrane permeability by measuring changes in virus size as a function of osmotic strength. The osmotic permeability of the viral membrane was characterized using SFLS by monitoring the change in light scattering intensity after rapidly mixing of inactivated influenza virus (diameter at ΔC_os_ = 0 mOsm is 105 ± 16 nm, see Fig A in S1 File) with high osmotic strength solutions. In SFLS, increased light scattering correlates with decreased virus size [41].

**Fig 1:**
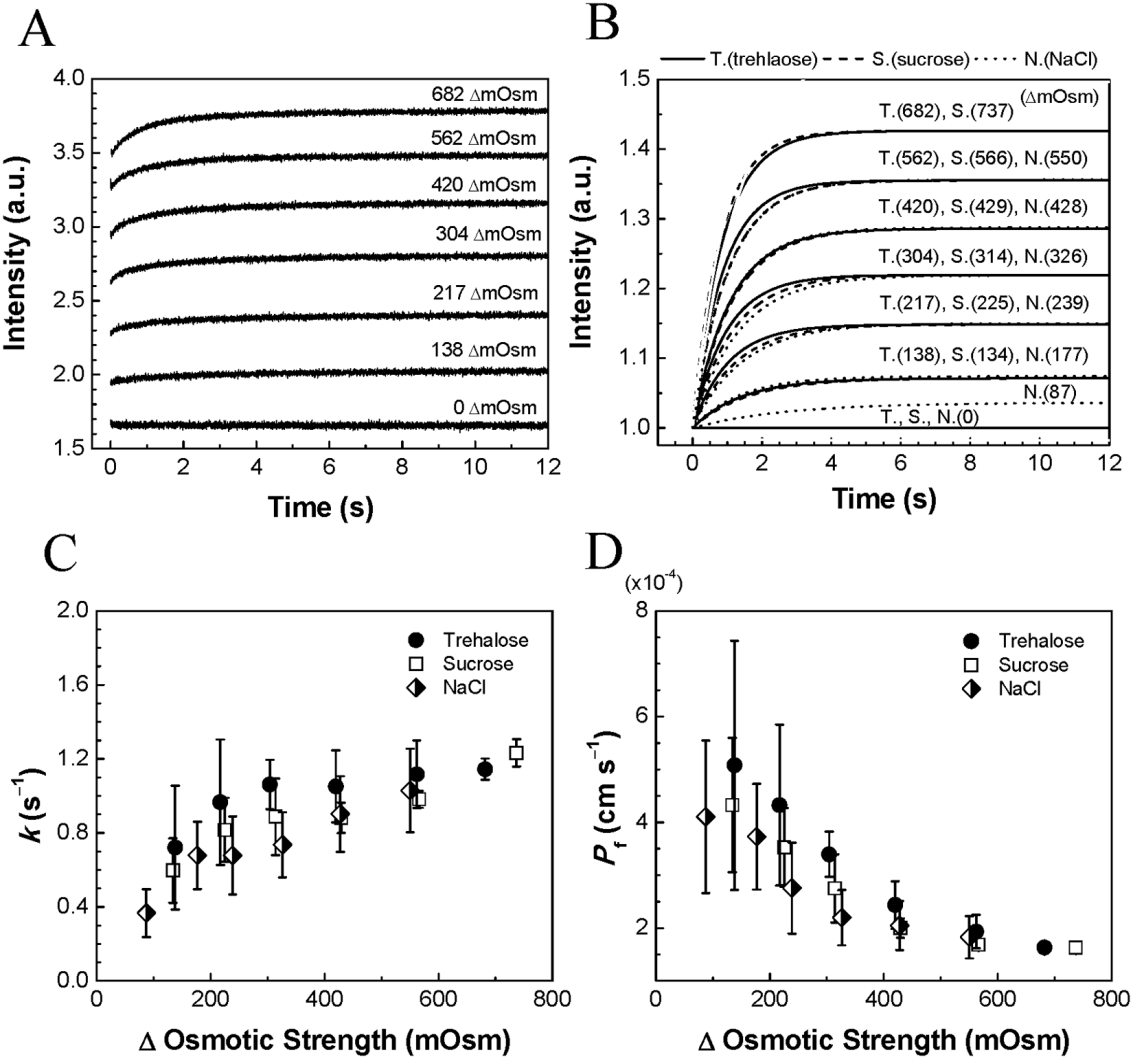
Time course of Stopped-flow light scattering (SFLS) analysis of inactivated influenza virus exposed to hypertonic solutions. A virus suspension (0.2 μg/μL) was abruptly exposed to hyperosmotic solutions of trehalose (*A*). Hyperosmotic viral shrinkage results in an increase of light scattering intensity, believed to be a result of water efflux from the virus. (*B*) Data in (*A*) were curve-fitted using single exponentials and plotted after normalization: trehalose (T), sucrose (S), NaCl (N). (*C*) Rate constants (*k* [s^–1^]) of the single exponential curves and (*D*) corresponding osmotic water permeability coefficients (*P*_f_ [cm s^–1^]). Data are presented as the mean ± standard deviation (SD) for *n* > 30. Hypertonic osmotic differences across the viral envelope (ΔC_os_) are indicated within the plots of (*A*) and (*B*).

The time-course of SFLS spectra were recorded for the first 12 s after exposing virus suspensions to hypertonic solutions of trehalose (Fig 1*A*), sucrose (Fig B(i) in S1 File), and NaCl (Fig B (ii) in S1 File). To test the effect of osmolytes on the leakage property of the virus, osmolarity of the external medium was controlled by changing the concentrations of the three osmolytes (trehalose, sucrose, and NaCl). The resulting data were fitted with single exponentials and corresponding curves are represented in Fig 1*B* after normalization.

As shown in Fig 1*A* and Fig B in S1 File, the influenza virus was found to exhibit typical osmotic behavior [37,42]. In the absence of an osmotic gradient (i.e., ΔC_os_ = 0 mOsm), no detectable level of SFLS intensity variation was observed from trehalose, sucrose, or NaCl solutions, indicating no change in virus size. In contrast, when exposed to outwardly directed, hyperosmotic gradients, viruses showed an abrupt increase in light scattering intensity (i.e., indicating shrinkage) followed by a slow saturation over time. Thus, the hyperosmotic gradient is thought to have triggered an efflux of water molecules out of the virus particles leading to a viral shrinkage with a rate dependant on the level of the applied hyperosmotic gradient.

Further examination of these data shows that the delay to saturation decreased as the osmotic differences across the viral membrane increased (Fig 1*B*). The shrinkage rate constant *k* increased in proportion to the osmotic strength imposed on the virus (Fig 1*C*). It should also be noted that NaCl exhibited slightly smaller *k* values than those of sucrose and trehalose solutions (ANOVA general linear model, *P* < 0.005), indicating the existence of a smaller osmotic difference across the viral membrane in the presence of NaCl than for sugar molecules. This can be explained by leakage of the viral membrane to small ions like Na^+^ and Cl^−^. As shown in Fig 1*D*, the osmotic water permeability *P*_f_ of the virus measured from all osmolytes ranged from 1×10^−4^ cm s^–1^ to 6×10^−4^ cm s^–1^ under the test conditions employed, which is similar to that of a lipid bilayer membrane [43].

**Fig 2:**
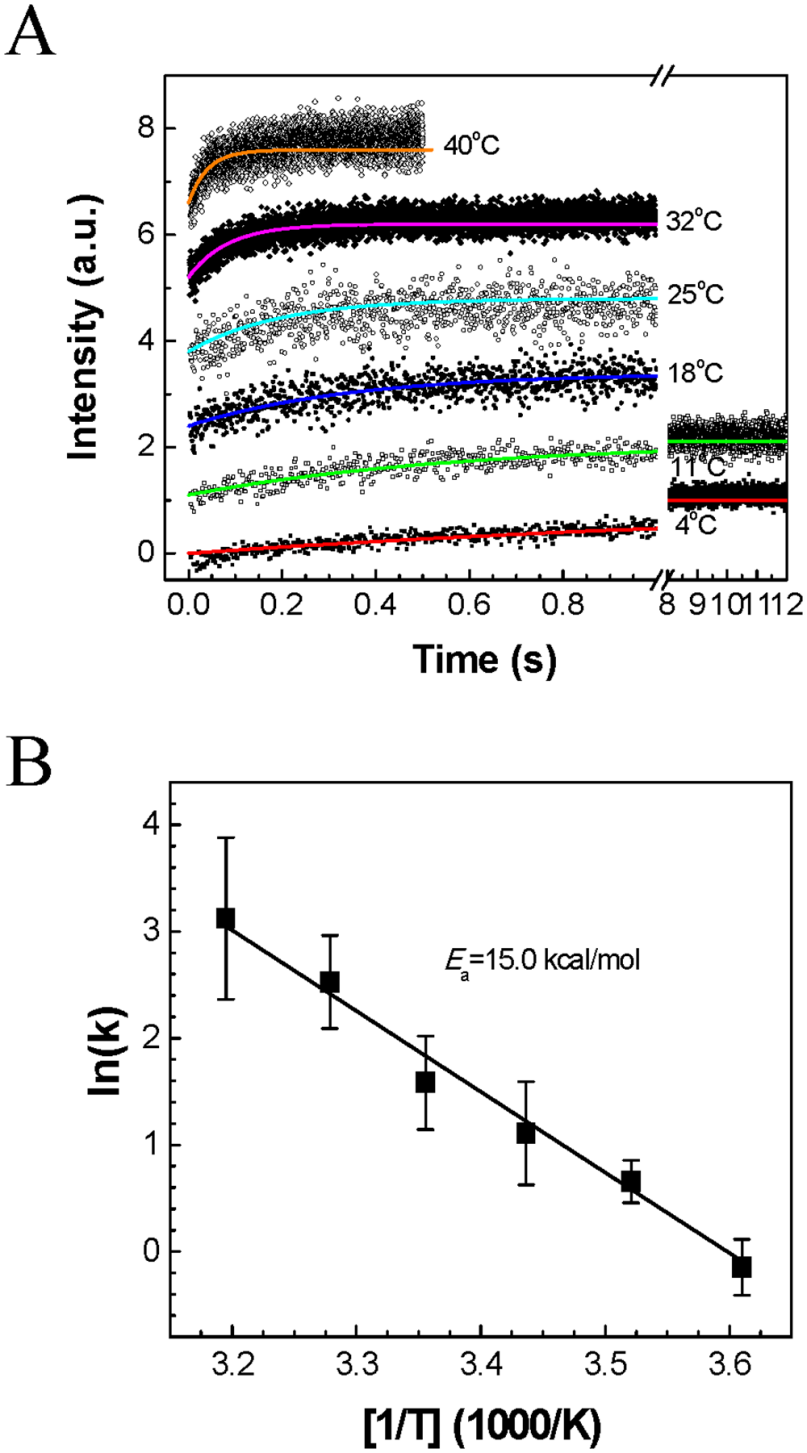
Temperature dependence of the osmotic response of inactivated influenza virus. The kinetics of osmotic water transport through the viral envelope were investigated at several temperatures while applying a hyperosmotic difference of ΔC_os_ = 304 mOsm with trehalose. (*A*) Scattered light intensities and fitted curves and (*B*) Arrhenius plot for water transport across the viral membranes (mean ± SD, *n* = 8–20). The Arrhenius activation energy was calculated from the linear regression of the ln(*k*) vs. 1/T plot.

The kinetics of osmotic water transport through the viral membrane was investigated at six different temperatures between 4°C and 40°C by applying a hyperosmotic difference of ΔC_os_ = 304 mOsm with trehalose across the viral membrane. Thermal fluctuations and instability in the virus envelope led to a noisy spectroscopic response upon hypertonic osmotic shock that showed greater variation at higher temperature (Fig 2*A*). Moreover, at higher temperature, the osmotic water permeability of the membrane significantly increased too (Fig C in S1 File). For example, a mean *P*_f_ of 3×10^−4^, 17×10^−4^, 45×10^−4^, and 92×10^−4^ cm s^–1^ was measured at 4, 25, 32, and 40°C, respectively. From the linear regression of the ln(*k*) vs. 1/T plot, a Arrhenius activation energy of 15.0 kcal mol^–1^ was calculated (Fig 2*B*). This is similar to the reported activation energy for water permeation across lipid bilayer membranes (10–17 kcal mol^–1^), as opposed to the much lower activation energy required for water transport through aqueous channel proteins (e.g., 3.7 kcal mol^–1^ for Aquaporin Z) [37,44]. This indicates that the pathway for water flow out of the virus during osmotically driven shrinkage was through the viral lipid membranes.

### Biphasic osmotic shrinkage behavior of inactivated influenza virus

**Fig 3:**
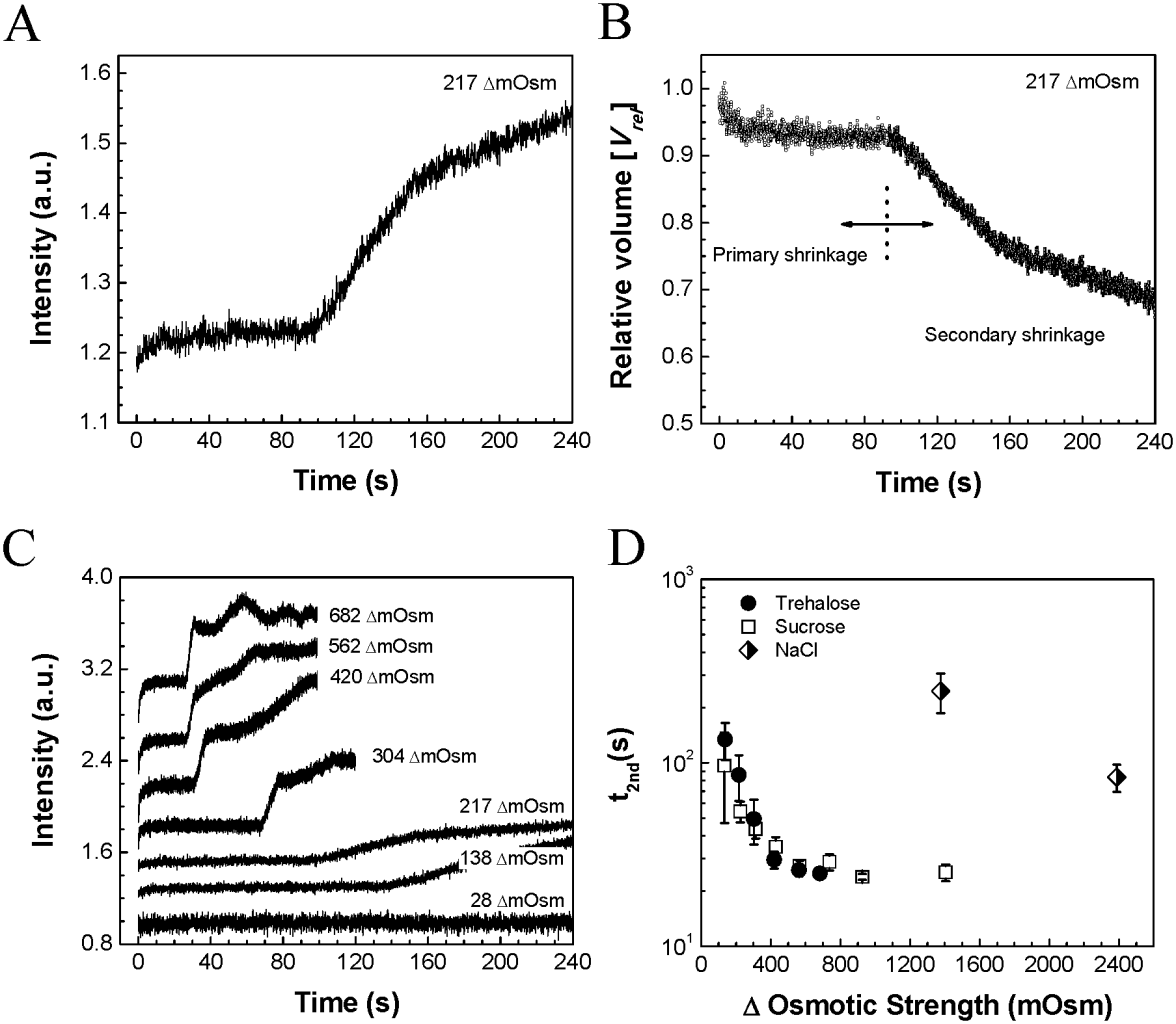
SFLS measurement of inactivated influenza virus. (*A*) Long-term course of SFLS behavior at an osmotic difference of ΔC_os_ = 217 mOsm trehalose and (*B*) the corresponding relative volume calculated as the initial volume divided by the volume at time *t*. (*C*) SFLS curves in response to hyperosmotic gradients in trehalose solutions. Hypertonic osmotic differences are indicated at the right side of each curve. The same test conditions were used as in Fig 1 except for an increased monitoring time. (*D*) The onset time for the secondary shrinkage (t_2nd_) as a function of osmotic gradients by trehalose, sucrose, and NaCl. (Mean ± SD, *n* = 9–19.)

To further investigate the morphological changes of the virus during osmotic stress, a long-term course of SFLS analysis was performed. Virus shrinkage was examined for 4 min under the same test conditions as in Fig 1. Fig 3*A* shows the SFLS data measured from inactivated virus exposed to osmotic strength difference of ΔC_os_ = 217 mOsm of trehalose. It is of interest to note that the SFLS curve exhibited a biphasic intensity increase over time. A steep increase in scattering intensity in the first few seconds (as seen in Fig 1) was followed by a plateau, which is attributed to the first shrinkage. About 100 s later, another SFLS intensity increase was observed, which had a steep slope for about 60 s and a shallower slope for at least another 80 s.

We translated these light scattering intensity changes into relative volumetric changes of the virus particles. This revealed that a rapid volumetric shrinkage down to about 93% of the initial volume occurred in the first phase, followed by a secondary shrinkage down to about 77% and then 69% of the initial total volume at 155 s and 240 s, respectively (Fig 3*B*). As indicated by the plateau on the SFLS curve (Fig 3*A*), it appears that osmotic shrinkage of the influenza virus proceeded in two separate phases (Fig 3*B*).

We carried out similar studies over a range of osmotic gradients generated by trehalose (Fig 3*C*), sucrose (Fig D(i) in S1 File), and NaCl (Fig D(ii) in S1 File) solutions. Both trehalose and sucrose solutions demonstrated similar osmotic responses. As seen in Fig 3*C* and Fig D(i) in S1 File, the viruses went through the previously observed biphasic morphological change at each of the osmotic strengths studied, although the time and degree of morphological change varied. The onset time for the secondary shrinkage (t_2nd_) decreased with the increase of osmotic stress (Fig 3*D*). It is important to note that at osmotic differences above ΔC_os_ = 682 mOsm, unstable intensity fluctuations were observed during or after the secondary shrinkage. This is important because that level of scattered light fluctuation can be explained by membrane destabilization. It appears that above a certain level of osmotic gradient, the lipids and matrix proteins (i.e., M1 proteins) comprising the envelope of the virus cannot adjust themselves to accommodate the stress during the secondary shrinkage phase, which results in the loss of membrane integrity and/or irreversible morphological change. This is discussed further below. In contrast, below this threshold osmotic gradient, secondary shrinkage can proceed without generation of significant or permanent membrane deformation. This explains why no notable level of intensity undulation was observed at ΔC_os_ = 28, 217, 304, and 420 mOsm osmotic strength after the secondary shrinkage. Therefore, we expect that the threshold stress above which viruses lose membrane integrity would be between ΔC_os_ = 562 and 682 mOsm.

These results, however, were not the same for a similar range of osmotic gradients caused by NaCl, which showed no such biphasic intensity increase (Fig D(ii) in S1 File). This can be explained by leakiness of the viral envelope to NaCl ions, which are much smaller than sugar molecules and can therefore cross the viral membrane more easily (Fig E in S1 File). Due to this dissipation of the osmotic gradient by membrane leakage, it is expected that significantly higher initial osmotic gradients are necessary to induce the secondary shrinkage of the virus in NaCl solutions (Fig 3*D*). Although the three osmolytes in this study exhibited different membrane leakage properties, it is reasonable to assume that effective osmotic stress is inversely related to t_2nd_. Thus, an osmotic difference of ΔC_os_ = 2388 mOsm by NaCl (t_2nd_ = 84 s) generated a similar level of effective osmotic difference to that of roughly 217 mOsm of trehalose (t_2nd_ = 86 s).

### Time-dependent functional hemagglutination activity of the virus

**Fig 4:**
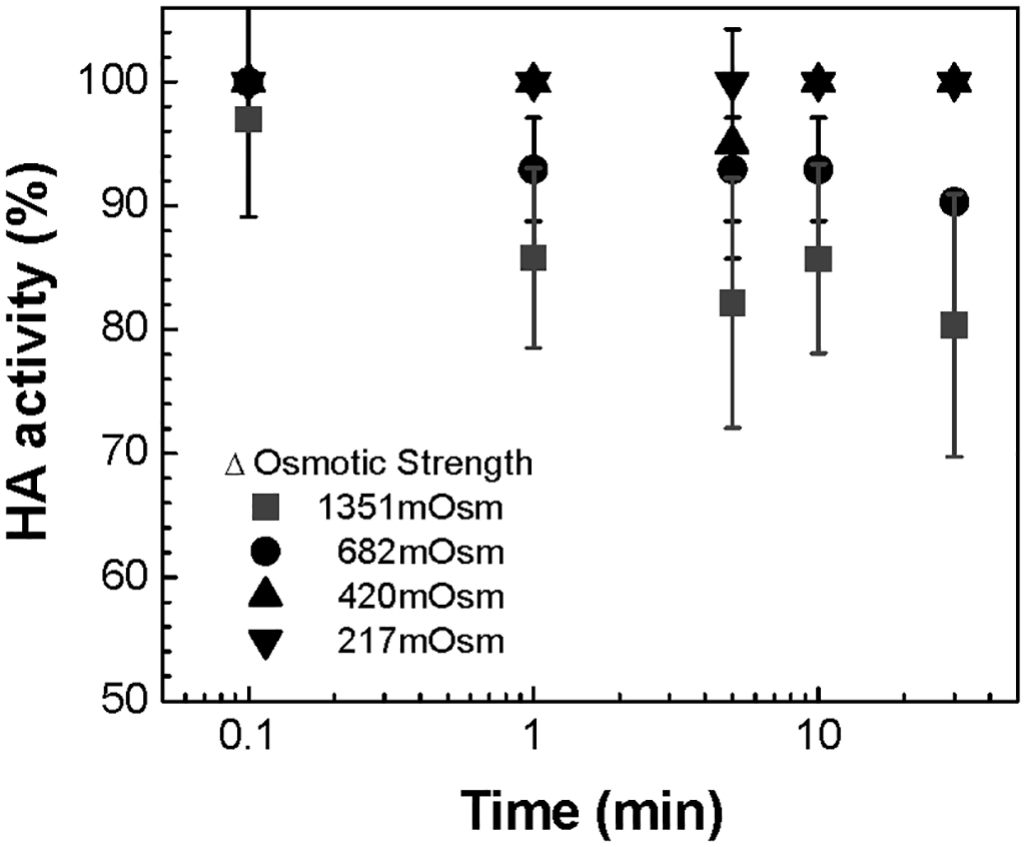
Hemagglutinin activity change as a function of incubation time and osmotic strength. The effect of osmotic pressures on the activity of the inactivated influenza virus was investigated by measuring HA activity change at four osmotic differences (ΔC_os_ = 217, 420, 682, 1351 mOsm) using trehalose with the increase of incubation time (10 s, 1 min, 5 min, 10 min, and 30 min). (Mean ± SD, *n* = 8–24.)

To evaluate the correlation between morphological change and viral activity, the functional activity of hemagglutinin in live and inactivated influenza virus was investigated at four osmotic conditions using trehalose. Functional hemagglutinin activity of the virus was assessed via HA assay as a function of incubation time in high osmotic strength solutions (Fig 4 and Fig F in S1 File).

The results in Fig 4 show that the inactivated virus began to show signs of activity loss within 1 min of exposure to an osmotic difference of at least ΔC_os_ = 682 mOsm, with an HA activity loss of about 10–20% after 30 min. The live virus reacted in a similar way, with a slight activity loss after the first minute followed by an increase in activity loss to about 10–20% after 5 min in response to osmotic differences of ΔC_os_ = 682 mOsm and 1351 mOsm, as shown in Fig F in S1 File. It should be noted that the activity loss at 1 min corresponds to the second phase of morphological change, where irregular scattering was observed at ΔC_os_ = 682 mOsm (Fig 3*C*). However, no significant viral activity loss was observed at ΔC_os_ = 217 mOsm or 420 mOsm, even after t_2nd_. For this reason, although systematic investigations on membrane deformation pattern were not performed in this work, we believe that the significant membrane perturbations caused by extreme osmotic stress, rather than the shrinking itself, could be the cause of the deactivation of the virus’ surface proteins, resulting in lower activity.

### Effect of viscosity on the stability of the influenza virus in solution

It is well known that increased viscosity decreases osmotic pressure [45,46]. Based on the expectation that integrity loss of the viral membrane during the secondary shrinkage is a key factor for vaccine stability, osmolyte solutions were modified to have higher viscosity with the goal of decreasing the extent of shrinkage and thus, minimizing membrane perturbation. In this work, CMC was used as a viscosity enhancer due to its expected safety as a widely used excipient in FDA-approved formulations (Fig G in S1 File for viscosity measurements). To evaluate this idea, stability of live and inactivated influenza virus in trehalose solution with and without CMC was examined.

**Fig 5:**
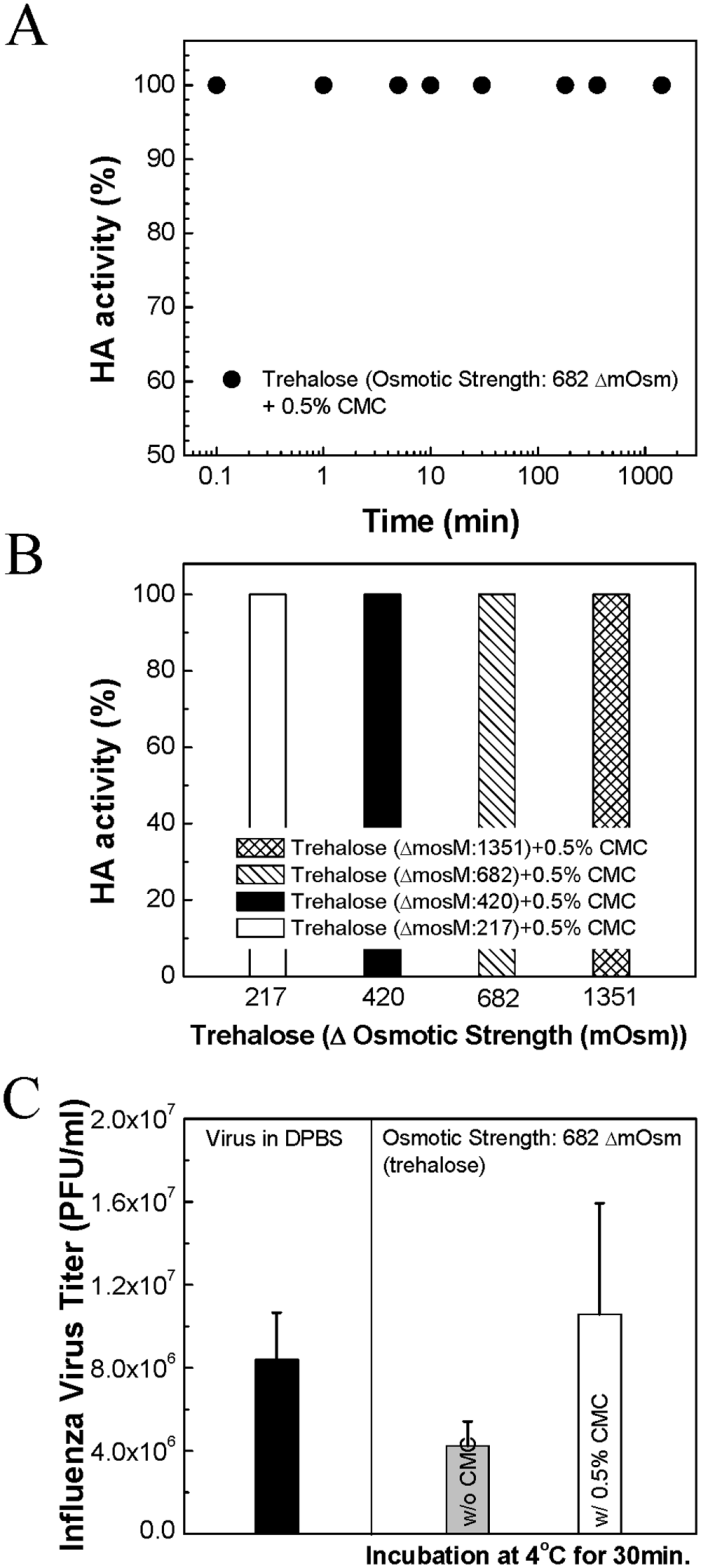
Effects of the viscosity enhancer CMC on the functional activity of the influenza virus. (*A*) Time-course of HA activity change of the inactivated influenza virus in a vaccine formulation composed of trehalose and CMC (*n* = 8). (*B*) HA activity as a function of osmotic differences (217, 420, 682, 1351 mOsm with trehalose) in the presence of 0.5% w/v CMC after incubation for 30 min (*n* = 8). (*C*) Viral titers of live influenza virus as assessed by plaque assay (*n* = 3). (Mean ± SD.)

Fig 5*A* shows the time-course of functional HA activity change of the inactivated virus in an osmolyte solution composed of trehalose (ΔC_os_ = 682 mOsm) and CMC (0.5% w/v). As shown in the plot, the virus did not show any significant activity loss, which contrasts with the results in the trehalose-only solution at the same concentration (Fig 4). Additional experiments over a range of trehalose concentrations incubated for 30 min at 4°C with CMC showed no functional HA activity drop, regardless of the osmotic strength contributed by trehalose (i.e., up to ΔC_os_ = 1351 mOsm) (Fig 5*B*).

In the case of live influenza virus, the effect of osmotic stress on its infectivity was investigated via plaque assay. For this purpose, viral titers of live influenza virus in both trehalose-only (ΔC_os_ = 682 mOsm) and trehalose (ΔC_os_ = 682 mOsm) plus CMC (0.5% w/v) solutions were assayed and compared to a control (i.e., virus in iso-osmotic solution of PBS). As expected from the experiment of live virus hemagglutinin activity loss in hypertonic solutions (Fig F in S1 File), a lower viral titer was measured from the trehalose-only solution than from the virus in iso-osmotic solution (Fig 5*C*). We also confirmed that the addition of CMC (0.5% w/v) resulted in a slower primary shrinkage (60% drop of *k*), a delay in the secondary shrinkage (1.8 fold increase of t_2nd_), and no irregular scattering after t_2nd_ compared with the trehalose-only solution (data not shown). These experiments indicate that the addition of a viscosity enhancer produces a dampening effect on the effective osmotic stress applied to the virus, thereby stabilizing viral membranes. The significance of this finding is that viscosity enhancers can play a critical role in stabilizing enveloped virus/vaccine under osmotic stress conditions.

### Effect of viscosity on the stability of influenza virus during drying

**Fig 6:**
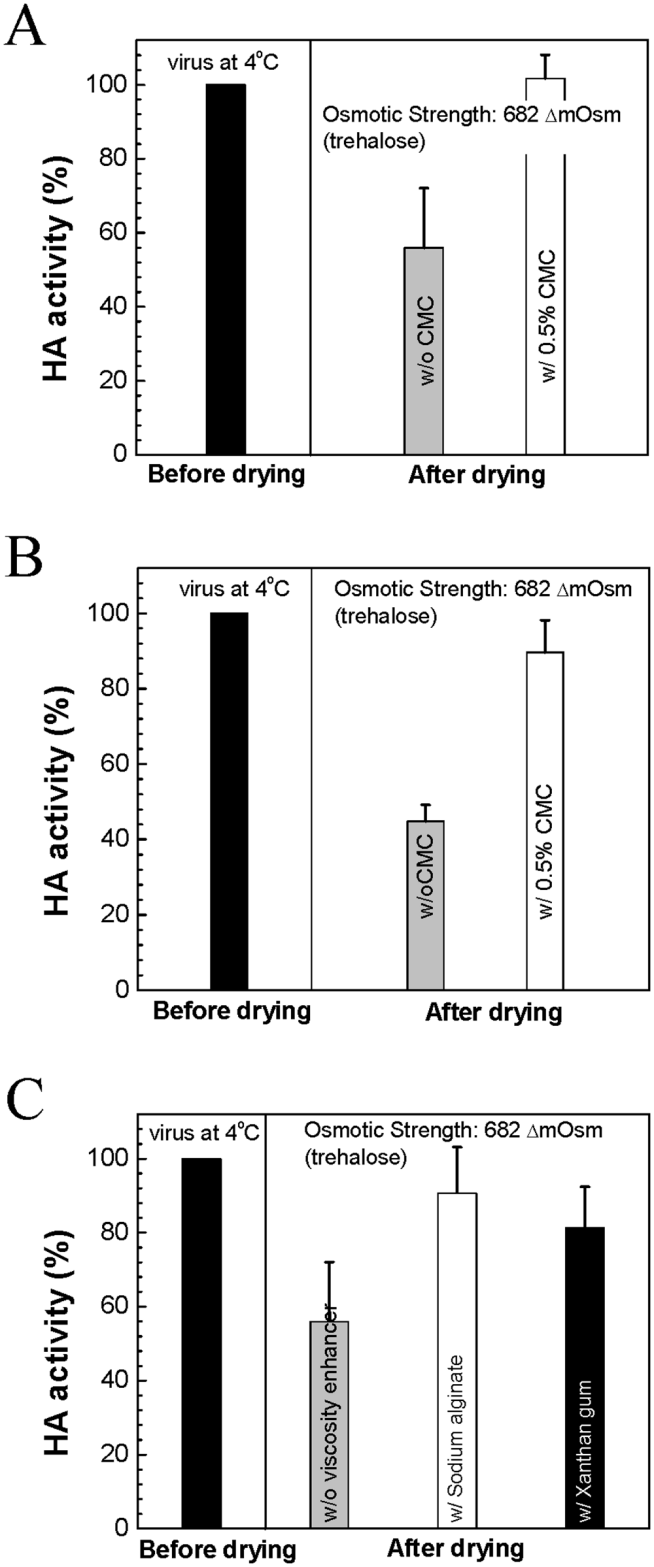
Effects of viscosity enhancer on the HA activity of influenza virus after drying. (*A*) Inactivated virus, (*B* **)** live virus, and (*C*) inactivated virus (mean ± SD, *n* = 8–16). In (*C*), HA activity of w/o viscosity enhancer was reused from (*A*) for better comparison.

Next, the stabilizing effects of a viscosity enhancer on the influenza virus were tested during drying. It is necessary to remember that during the drying process, osmotic stress increases with the progress of dehydration. After one day drying at ambient conditions, functional HA activity change was measured both in the presence and absence of CMC using live and inactivated influenza virus (Fig 6*A*: inactivated virus, Fig 6*B*: live virus). HA titers of each virus type in iso-osmotic solution were used as a reference. As shown in Fig 6*A* and 6*B*, while viruses with the trehalose (ΔC_os_ = 682 mOsm) plus CMC (0.5% w/v) solution maintained most of their original activity even after drying (100% for inactivated virus, 90% for live virus), only 56% and 45% of the remaining HA activity were measured with the trehalose-only (ΔC_os_ = 682 mOsm) solution from the inactivated and live viruses, respectively (Student's *t*-test, *P* < 0.001).

The stabilizing effect of the viscosity increase was further investigated using the trehalose solutions with different viscosity enhancers, but with the same viscosity as the trehalose (ΔCos = 682 mOsm) plus CMC (0.5% w/v): i.e., trehalose (ΔC_os_ = 682 mOsm) plus xanthan gum (0.075% w/v) and trehalose (ΔC_os_ = 682 mOsm) plus sodium alginate (0.3% w/v). As shown in Fig 6*C*, the presence of viscosity enhancer exhibited significantly higher level of HA activity than the trehalose only (91% for sodium alginate, 81% for xanthan gum) (Student's *t*-test, *P* < 0.001). These results further support the hypothesis that a viscosity enhancer can stabilize enveloped virus against osmotic stress by suppressing membrane perturbation-induced defunctionalization of antigenic proteins.

### *In vivo* experiments using influenza-vaccine coated MNs

To confirm previous *in vitro* findings, *in vivo* experiments were performed using two different kinds of inactivated influenza virus-coated MNs: virus coating with the trehalose-only (ΔC_os_ = 682 mOsm) formulation (Fig H(i) in S1 File) and virus coating with the trehalose (ΔC_os_ = 682 mOsm) plus CMC (0.5% w/v) formulation (Fig H(ii) in S1 File). Coated MN arrays of five needles each (Fig H in S1 File, (i) and (ii) insets) were air-dried for one day at ambient conditions (23°C, 30–60% relative humidity). Vaccine coatings were then resuspended in iso-osmotic solution and given by intramuscular injection to vaccinate mice. *In vivo* experiments were performed using four different groups of mice: naïve (negative control), virus in iso-osmotic solution (positive control), solution reconstituted from dried vaccine coating on MN (trehalose-only), and solution reconstituted from dried vaccine coating on MN (trehalose plus CMC). Vaccine from MN coatings was reconstituted and given by injection rather than being administered directly using the MNs in order to remove the effects of the intradermal route of administration using solid MNs and thereby provide a more direct comparison among liquid formulations injected intramuscularly.

**Fig 7:**
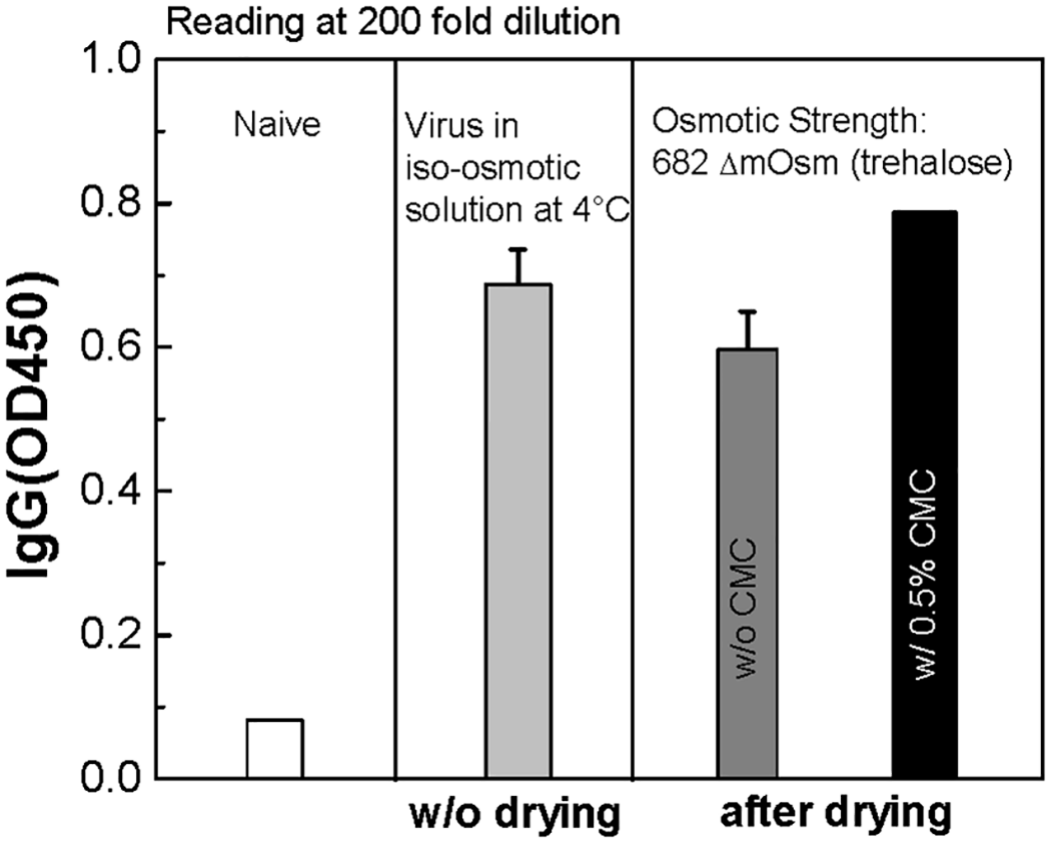
Effect of dried formulations of vaccine on *in vivo* immunogenicity. Influenza vaccine-coated MNs were air-dried for one day at ambient conditions and reconstituted in DPBS for intramuscular vaccination of mice (*n* = 6 per group). Naïve (negative control), vaccine in iso-osmotic solution (positive control), dried vaccine-coated MNs (w/o CMC: trehalose-only, w/ 0.5% w/v CMC: trehalose plus CMC). Virus-specific IgG was assayed by ELISA with the immune sera of mice two weeks after vaccination with 0.2 μg of viral proteins. Optical density was measured at 450 nm. (Mean ± SD, *n* = 6.)

To evaluate the immunogenicity of these four groups, virus-specific IgG antibody levels were determined by ELISA from the sera of mice two weeks after vaccination with 0.2 μg of viral proteins. As shown in Fig 7, a lower level of influenza virus-specific antibody production was observed from the trehalose-only group than the positive control group. On the other hand, the trehalose-plus-CMC group showed a similar level of antibody production to the positive control group, and higher levels of antibodies compared to the trehalose-only group. The high levels of IgG antibodies observed from the influenza vaccine-coated MNs with the trehalose-plus-CMC formulation further support our previous finding that the addition of viscosity enhancer in the vaccine formulation reduces osmotic stress-induced vaccine activity loss during drying. The results of this study suggest that maintaining a high viscosity during drying is important for maintaining vaccine stability.

## Discussion

Vaccine-coated MNs have been proposed as a promising vaccine administration method that simplifies vaccination and has the potential to thereby increase vaccination coverage. This led us to investigate destabilizing factors on influenza vaccine-coated MNs and their associated mechanisms. Similar to other enveloped organisms, osmotic pressure is an important factor affecting the stability of influenza viral vaccines. Unlike bacteria or other cells, there is an inherent lack of systematic study on viral osmotic behavior. This makes it difficult to predict the stability of viral vaccines under various environmental conditions, and to identify solutions to stability problems. Therefore, this study deals with conditions under which enveloped virus becomes damaged under osmotic stress and proposes a strategy to avoid osmotic pressure-induced stability loss when making influenza vaccine-coated MNs.

Inactivated influenza virus was used as a model system to investigate osmotic stability of enveloped viruses. Our first goal was to characterize osmotic water leakage of the viral envelope via SFLS analysis using trehalose, sucrose, and NaCl as osmolytes. Under a hypertonic osmotic gradient, at the initial shrinkage phase (i.e., < 4 s), the virus’ shrinking behavior was similar to other enveloped biological systems, as determined by scattered light intensity as a measure of viral volume change. At an osmotic difference of ΔC_os_ = 304 (trehalose), 314 mOsm (sucrose), and 326 mOsm (NaCl), osmotic water permeability was measured to be 3.4 ± 0.4×10^−4^ (trehalose), 2.7 ± 0.6×10^−4^ (sucrose), and 2.2 ± 0.5×10^−4^ (NaCl) cm/s, with a high activation energy (*E*_a_ = 15.0 kcal mol^–1^). These measurements are similar to those previously found for pure liposomes without any osmoregulatory proteins, indicating that only pathway for water transport is through the membrane lipids [44].

The most significant finding from the long-term measurements by SFLS with trehalose and sucrose is that the virus experienced biphasic osmotic shrinking behavior under hypertonic osmotic conditions. As demonstrated by the biphasic increase of scattered light intensity, a gradual secondary shrinkage over the course of tens of seconds to minutes was observed to follow a rapid primary shrinkage. For the NaCl osmolyte solution, a much higher level of osmolarity was required to observe the secondary shrinkage than sucrose/trehalose, believed to be due to leakage of small Na^+^ and Cl^−^ ions through the viral membrane. Theses SFLS results observed over a range of experimental conditions and seen in both live and inactivated virus indicate that this biphasic pattern represents a characteristic osmotic shrinking behavior of influenza virus in response to hyperosmotic gradients. Furthermore, from the relationship between osmotic gradient and t_2nd_, we hypothesize that t_2nd_ can be used to estimate the effective osmotic gradient applied to the virus, regardless of osmolytes.

To further understand this osmotic shrinking behavior, we should consider the viral structure. An influenza virus contains two outer layers: a lipid bilayer (~ 3.8 nm thick) and a matrix protein (M1) layer (~ 7.2 nm thick) [47,48]. The outer surface of the virus consists of a lipid envelope where antigenic glycoproteins such as hemagglutinin and neuraminidase (NA), and ion channel proteins (M2) are embedded. The inner surface is lined by the M1 protein (27 kDa), which has been thought to provide mechanical protection to the virus, similar to viral capsids [49,50] and determine the morphology of the virus through the interactions with lipids, glycoproteins, and the ribonucleoprotein complex (RNP) [47,51–56]. Although the compressibility for M1 is unknown, the volumetric compressibility of globular proteins have been reported to exhibit 1–2 orders of magnitude smaller than that of liquid crystalline lipid bilayers [57]. From a mechanical property point of view, these two viral layers can be viewed as a composite structure with a flexible lipid bilayer membrane supported internally by a shell of M1. As a consequence, the shrinking kinetics of the virus is largely determined by physical features of these two layers.

We hypothesize that the presence of biphasic morphological changes could be attributed to this double-shell structure. Upon exposure to hyperosmotic shock, an efflux of water molecules from the virus causes the viral envelope to shrink, which is expected to increase lateral packing density of both lipid molecules in the lipid bilayer and protein molecules in supporting the M1 layer. These layers will compress easily to a certain degree, as reflected by the initial rapid change in viral volume followed by a quasi-steady state indicated by the plateau seen in the SFLS spectra. This resistance to further shrinkage can be explained mainly by the resistance of the M1 protein layer to further compression. In a previous study by Ivanovska et al., bacteriophage capsids have been found to exhibit bimodal elastic properties [49]. We are uncertain about the quantitative contribution of the glycoprotein-embedded lipid membrane and M1 shell to the morphological change. Additionally, the effect of structural change due to formaldehyde inactivation on the shrinkage process was not investigated in this work. Without M1, the virus would be almost the same as a liposome, except for the glycoproteins.

When the compressive stress exceeds a tolerance level during and/or after the secondary shrinkage, membrane integrity is lost, and further deformation of the virus is expected to occur in order to relieve the stress being applied to it. The membrane perturbation/morphological change would result in highly undulating light scattering intensity profile, which explains SFLS spectra of the influenza virus during strong osmotic shock (ΔC_os_ = 682 mOsm for trehalose and 737 mOsm for sucrose) as shown in Fig 3*C* and Fig D(i) in S1 File.

The effects of structural differences among biological membranes on shape change have been studied in the past using liposomes filled with polymer gels [58,59] or actin polymers [60–63] to mimic the composite membrane structure of eukaryotic cells. Viallat et al. have shown that gel-filled liposomes developed a spike-like morphology under hyperosmotic conditions [58]. More importantly, in the case of a slight deformation, upon re-exposure to iso-osmotic conditions, the liposomes were found to swell and the spike-like protrusions disappeared. However, significantly deformed liposomes (to about 80% of initial volume) were not able to completely recover their morphology even after the re-swelling process. Despite the possible difference in the ultimate osmotic stress between liposome and influenza virus, such an irreversible membrane deformation provides a reasonable explanation for the undulating SFLS profile following the biphasic viral shrinkage. This is also in agreement with our hypothesis describing the osmotic stress-induced perturbation of the viral membranes.

We also concentrated on studying the relationship between membrane deformation and viral activity loss by means of HA titers and viral plaque assay. As demonstrated in Fig 4 and Fig F in S1 File, both live and inactivated influenza viruses exhibited activity loss at the time points corresponding to membrane deformation in the SFLS spectra. HA activity decreased by about 10–20% after incubation for 30 min in hyperosmotic solution. The large errors in HA activity at 1351 ΔmOsm for inactivated virus (Fig 4) and the apparent decrease in HA activity at 682 ΔmOsm compared to 1351 ΔmOsm for live virus (Fig F in S1 File) can be explained by the unpredictable pattern of membrane deformation at high hyperosmotic stresses as observed from SFLS analyses. Consequently, we conclude that irreversible membrane deformation during and/or after the secondary shrinkage are associated with viral activity loss. During deformation, a certain degree of membrane distortion can occur, and the lipids are stretched or compressed depending on the curvature of the membranes. According to previous reports, a variation of lipid length can generate hydrophobic mismatch and curvature stress, which has been successfully used in predicting the conformation, function, and lifetime of proteins and peptides [57,64–69]. Considering the fact that antigenic proteins (i.e., hemagglutinin) reside in the membrane in order to maintain their functional integrity, we propose that mechanical perturbation of the membrane increases the probability of imposing locally high stress to the proteins, thereby inducing irreversible conformational changes to the proteins. In spite of differences in both the membrane structure and osmotic conditions, this finding shares a similarity with Gram-negative bacteria in osmotic pressure-induced loss of wall integrity and thus, the resulting viability loss [24,58,70–72].

As a way to prevent osmotic stress-induced viral activity loss, we tested the effect of viscosity on viral stability. The data showed that time-dependant osmotic shrinkage was significantly inhibited by the presence of a viscosity enhancer. In other words, the viscosity enhancer played a critical role in reducing osmotic pressure-mediated effects on the virus. As a result, the effective osmotic gradients which would cause the virus to become destabilized under low-viscosity conditions were reduced to below destabilizing levels. For that reason, no loss in viral activity (HA titer, virus infectivity) was observed even at ΔC_os_ = 682 mOsm and 1351 mOsm in the osmolyte solution containing viscosity enhancer (CMC in trehalose).

Another effect of viscosity enhancer is on the stabilization of viral vaccines during drying. It is well known that osmotic pressure increases as water content decreases through dehydration. The resulting increased stress is a major destabilization factor for biomolecules (e.g., in vesicles, cells, vaccines, etc.). Therefore, controlling osmotic stress-induced destabilization is of special interest to scientists working in the area of designing drug formulations (liquid, solid) and devising drying methods. As seen in the liquid state, we similarly observed a stabilizing effect on both live and inactivated influenza viruses in the post-drying, solid state when a viscosity enhancer was added (see Fig 6). Importantly, the same stabilizing effects were observed when different viscosity enhancers, such as sodium alginate and xanthan gum, were added instead. This indicates that the vaccine stabilization is due to the viscosity increase, not just CMC, and could be a general feature of viscosity-enhanced vaccine formulations. These results are important because controlling viscosity in a formulation is a simple means to overcome osmotically driven destabilization during drying that does not require developing new drying methods or protocols.

As a result, solid MNs coated with influenza virus using the trehalose-plus-viscosity enhancer formulation were shown to maintain their original antigenic immunogenicity even after 24 h of drying. This indicates that our *in vitro* findings about the effects of osmotic stress on viral stability could be generalized from the liquid state to the more important solid state. Although our research was performed only on influenza virus, we propose that the general idea of osmotic pressure-induced viral deactivation and the stabilizing effect of a viscosity enhancer can be used in the future development of both solid and liquid drug formulations for other enveloped vaccines, as exemplified by influenza vaccine-coated MNs.

## Conclusion

In this work, SFLS analysis has been employed in monitoring the time-dependent structural stability of an enveloped influenza virus under various osmotic test conditions and in developing MN vaccine formulations. We found that disaccharide-induced osmotic stress generated biphasic morphological change of the influenza virus. The onset time for the secondary shrinkage/morphological change and the degree of undulation in scattered light intensity provided quantitative/qualitative information about the effective osmotic stress applied to the virus and the structural integrity of the virus envelope, respectively. The presence of a viscosity enhancer in the solution was shown to protect virus from osmotic damage both in liquid and in dried states, by means of *in vitro* and *in vivo* experiments. Overall, our results suggest that enveloped influenza virus undergoes biphasic shrinkage under hyperosmotic conditions that can lead to loss of virus immunological activity. The addition of a viscosity enhancer (CMC) in a hyperosmotic sugar-based vaccine formulation was shown to inhibit this osmotically driven damage and therefore is a useful excipient in dried-vaccine coating formulations on MNs.

## Supporting Information

S1 File **Fig A** Transmission electron microscopy image of inactivated influenza virus. As shown in the micrograph, viruses are spherical in shape and spike-shaped glycoproteins are observed on the surface of the viral envelope. A mean diameter of the virus was measured to be 105 ± 16 nm from the analysis of such micrographs. **Fig B** Time course of SFLS analysis of inactivated influenza virus exposed to hypertonic solutions. Virus suspensions (0.2 μg μL^−1^) were abruptly exposed to hyperosmotic solutions of (i) sucrose and (ii) NaCl in a stopped-flow apparatus and the resulting changes in scattered light intensity were monitored at 546 nm as a function of time for 12 s. All measurements were made at 4°C. Data are presented as the mean ± standard deviation (SD) for *n* > 30. Hypertonic osmotic differences (ΔC_os_) are indicated within the plots of (i) and (ii). **Fig C** Temperature dependence of the osmotic water permeability coefficients of inactivated influenza virus derived from data in Fig 2. (Mean ± SD, *n* = 15–30.) **Fig D** Long-term course of SFLS behavior of inactivated influenza virus in response to hyperosmotic gradients by (i) sucrose and (ii) NaCl solution. Hypertonic osmotic differences (ΔC_os_) are indicated at the right side of each curve. **Fig E** Long-term course of SFLS analysis of inactivated influenza virus in response to a hyperosmotic difference of ΔC_os_ = 239 mOsm by NaCl solution. A biphasic intensity increase was not observed from the virus with NaCl solution at this condition. This can be explained by the leakage of the viral envelope to Na^+^, Cl^−^ ions, as indicated by a gradual intensity decrease following the first phase of rapid intensity increase. **Fig F** HA activity change as a function of incubation time in hypertonic solutions. The effect of osmotic pressure on the activity of live influenza virus was investigated by measuring HA activity change at four osmotic strength differences (ΔC_os_ = 217, 420, 682, 1351 mOsm) using trehalose with the increase of incubation time (10 s, 1 min, 5 min, 10 min, and 30 min). HA activity change was calculated with respect to HA titer of the virus in iso-osmotic solution. All measurements were performed at 4°C. (Mean ± SD, *n* = 8–16.) **Fig G** Viscosity of the trehalose (ΔC_os_ = 682 mOsm) solution and the trehalose (ΔC_os_ = 682 mOsm) plus CMC (0.5% w/v) solution. (Mean ± SD, *n* = 3.) **Fig H** Dried vaccine-coated MNs with inactivated influenza virus in formulations of (i) trehalose (ΔC_os_ = 682 mOsm) only and (ii) trehalose (682 mOsm) plus viscosity enhancer CMC (0.5% w/v). Influenza vaccine-coated MNs were air-dried for one day at ambient conditions and reconstituted in DPBS for vaccination of mice.
(PDF)

S1 Dataset (XLSX)
