# Supplementary material for: Effect of Osmotic Pressure on the Stability of Whole Inactivated Influenza Vaccine for Coating on Microneedles
Source: PLoS One. 2015 Jul 31;10(7):e0134431. doi: 10.1371/journal.pone.0134431 (PMC4521748; doi:10.1371/journal.pone.0134431)
Supplement: S1 File — Fig A Transmission electron microscopy image of inactivated influenza virus. As shown in the micrograph, viruses are spherical in shape and spike-shaped glycoproteins are observed on the surface of the viral envelope. A mean diameter of the virus was measured to be 105 ± 16 nm from the analysis of such micrographs. Fig B Time course of SFLS analysis of inactivated influenza virus exposed to hypertonic solutions. Virus suspensions (0.2 μg μL−1) were abruptly exposed to hyperosmotic solutions of (i) sucrose and (ii) NaCl in a stopped-flow apparatus and the resulting changes in scattered light intensity were monitored at 546 nm as a function of time for 12 s. All measurements were made at 4°C. Data are presented as the mean ± standard deviation (SD) for n > 30. Hypertonic osmotic differences (ΔCos) are indicated within the plots of (i) and (ii). Fig C Temperature dependence of the osmotic water permeability coefficients of inactivated influenza virus derived from data in Fig 2. (Mean ± SD, n = 15–30.) Fig D Long-term course of SFLS behavior of inactivated influenza virus in response to hyperosmotic gradients by (i) sucrose and (ii) NaCl solution. Hypertonic osmotic differences (ΔCos) are indicated at the right side of each curve. Fig E Long-term course of SFLS analysis of inactivated influenza virus in response to a hyperosmotic difference of ΔCos = 239 mOsm by NaCl solution. A biphasic intensity increase was not observed from the virus with NaCl solution at this condition. This can be explained by the leakage of the viral envelope to Na+, Cl− ions, as indicated by a gradual intensity decrease following the first phase of rapid intensity increase. Fig F HA activity change as a function of incubation time in hypertonic solutions. The effect of osmotic pressure on the activity of live influenza virus was investigated by measuring HA activity change at four osmotic strength differences (ΔCos = 217, 420, 682, 1351 mOsm) using trehalose with the increase of incubation time [file pone.0134431.s001.pdf]

Supporting Information 1

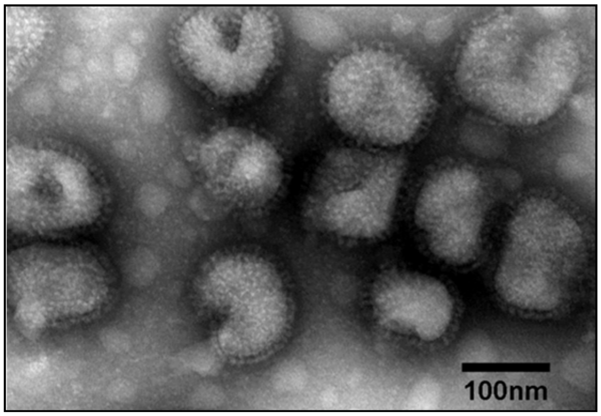

S1 Fig. A Transmission electron microscopy image of inactivated influenza virus. As shown in the micrograph, viruses are spherical in shape and spike-shaped glycoproteins are observed on the surface of the viral envelope. A mean diameter of the virus was measured to be 105 ± 16 nm from the analysis of such micrographs.

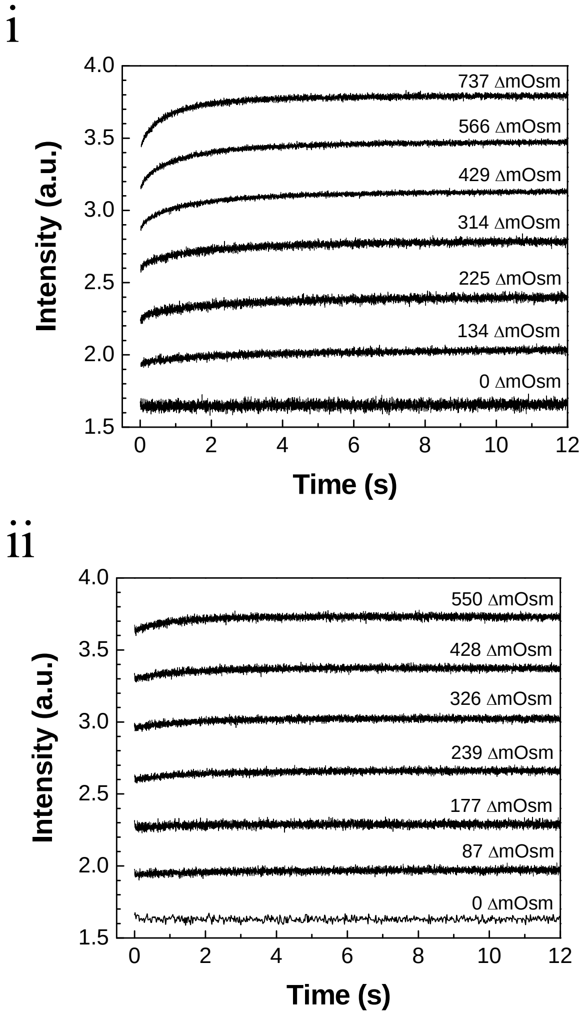

S1 Fig. B Time course of SFLS analysis of inactivated influenza virus exposed to hypertonic solutions. Virus suspensions (0.2 μg μL^{-1}) were abruptly exposed to hyperosmotic solutions of (i) sucrose and (ii) NaCl in a stopped-flow apparatus and the resulting changes in scattered light intensity were monitored at 546 nm as a function of time for 12 s. All measurements were made at 4 °C. Data are presented as the mean ± standard deviation (SD) for $n > 30$. Hypertonic osmotic differences (ΔC_{os}) are indicated within the plots of (i) and (ii).

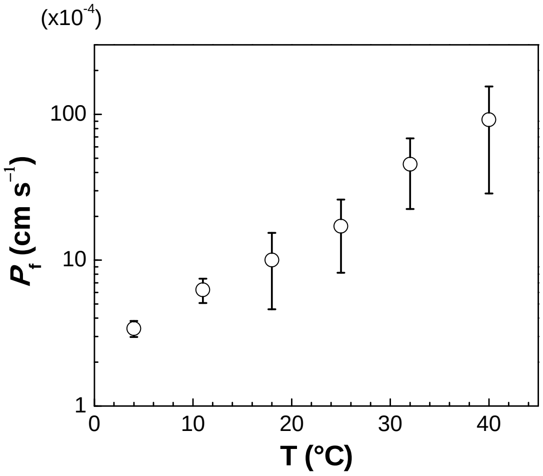

S1 Fig. C Temperature dependence of the osmotic water permeability coefficients of inactivated influenza virus derived from data in Fig. 2. (Mean ± SD, n = 15 – 30.)

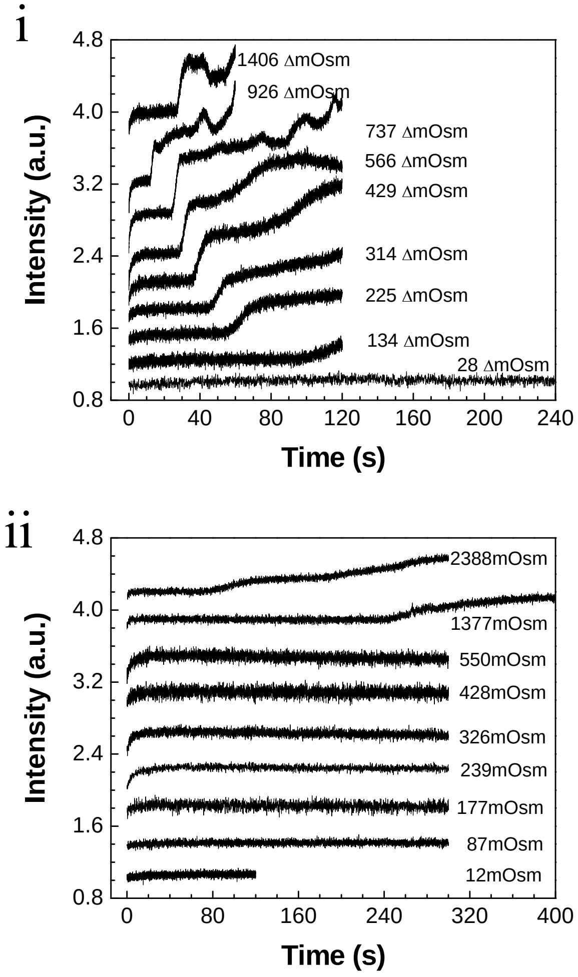

S1 Fig. D Long-term course of SFLS behavior of inactivated influenza virus in response to hyperosmotic gradients by (i) sucrose and (ii) NaCl solution. Hypertonic osmotic differences (ΔC_{os}) are indicated at the right side of each curve.

Supporting Information 1

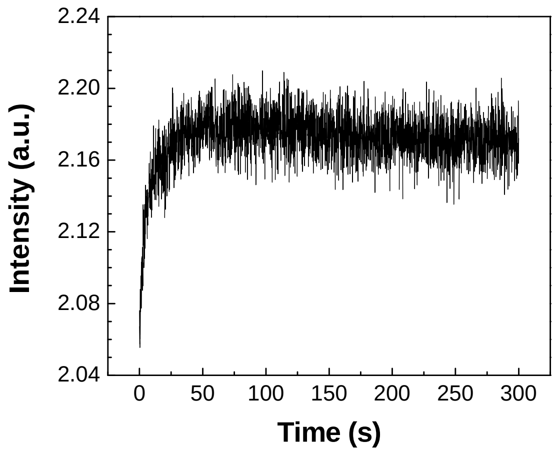

S1 Fig. E Long-term course of SFLS analysis of inactivated influenza virus in response to a hyperosmotic difference of ΔC_{os} = 239 mOsm by NaCl solution. A biphasic intensity increase was not observed from the virus with NaCl solution at this condition. This can be explained by the leakage of the viral envelope to Na^+ Cl^- ions, as indicated by a gradual intensity decrease following the first phase of rapid intensity increase.

Supporting Information 1

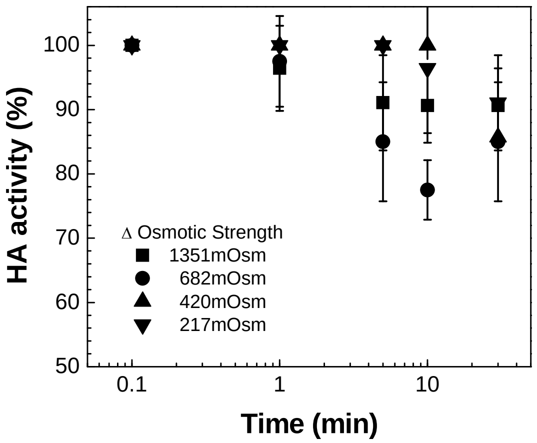

S1 Fig. F HA activity change as a function of incubation time in hypertonic solutions. The effect of osmotic pressure on the activity of live influenza virus was investigated by measuring HA activity change at four osmotic strength differences (ΔC_{os} = 217, 420, 682, 1351 mOsm) using trehalose with the increase of incubation time (10 s, 1 min, 5 min, 10 min, and 30 min). HA activity change was calculated with respect to HA titer of the virus in iso-osmotic solution. All measurements were performed at 4 °C. (Mean ± SD, *n* = 8 – 16.)

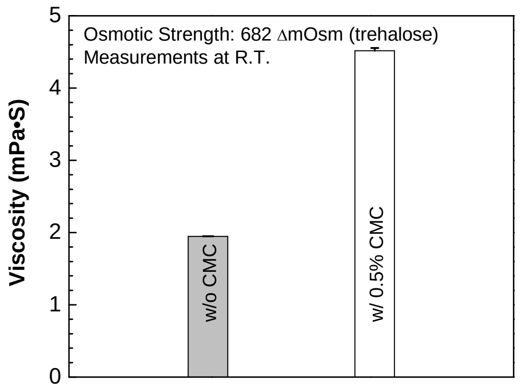

S1 Fig. G Viscosity of the trehalose (ΔC_{os} = 682 mOsm) solution and the trehalose (ΔC_{os} = 682 mOsm) plus CMC (0.5% w/v) solution. (Mean ± SD, *n* = 3.)

Supporting Information 1

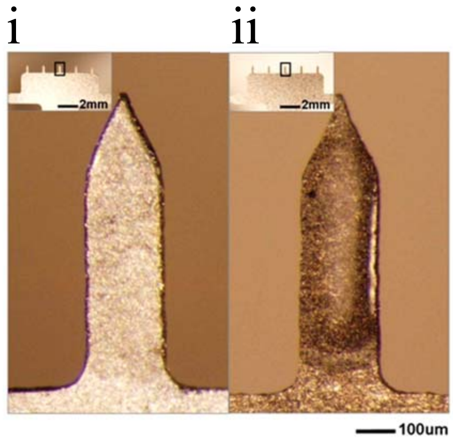

S1 Fig. H Dried vaccine-coated MNs with inactivated influenza virus in formulations of (i) trehalose (ΔC_{os} = 682 mOsm) only and (ii) trehalose (682 ΔmOsm) plus viscosity enhancer CMC (0.5% w/v). Influenza vaccine-coated MNs were air-dried for one day at ambient conditions and reconstituted in DPBS for vaccination of mice.
